# Beer Drinking

**Published:** 1892-07

**Authors:** 


### BEER DRINKING.

A notion still lingers in the minds of many, says Professor M'Intosh, M.D., in an address at Edinburgh, that beer and even alcohol are required in the dietary of an ordinarily healthy individual. They may, perhaps, be imagined to be necessary, in the same way that the fancied invalid daily doses himself with bitters and stomachics to improve his digestion, while he loads the stomach with too much and often too indigestible food. It is true that small quantities of beer or alcohol, taken on rare occasions, may exhibit no appreciable effect of a permanent kind; but neither do small doses of prussic acid or arsenic taken in a similar way. This mode of testing is, therefore, no reliable proof of the harmlessness of either. The brewer's man, on the other hand, who habitually imbibes beer, is a conspicuous example of its deleterious influence, though perhaps he may never have been known to exhibit those features of poisoning which we call intoxication. Yet a slight wound or abrasion on any part of the body is prone to take on an unhealthy action, and he perhaps succumbs to an injury which would have been comparatively trifling in a man who does not imbibe beer. The surgeon well knows how unfavorable a type the brewer's man is for any—even the slightest—surgical operation; while the physician is likewise familiar with the rapid and fatal internal changes characteristic of the same type of patient. This shows that the habitual use of the apparently harmless beer brings the system into an unhealthy condition; and I am sure I need hardly tell you that the relationship existing between body and mind is so close that the one acts and reacts on the other. Moreover, there is no part of the organism which becomes sooner affected under these circumstances than the nervous system. Hence the beer-drinker is less favorably situated intellectually than a man of similar calibre who abstains from the apparently innocuous beverage.

